# Glycolysis enzymes and cellular lactylation in tumour

**DOI:** 10.1002/ctm2.70549

**Published:** 2026-02-18

**Authors:** Chenyuan Dai, Lihua Wang

**Affiliations:** ^1^ Department of Cellular and Genetic Medicine School of Basic Medical Sciences Fudan University Shanghai China

**Keywords:** glycolysis enzymes, lactylation, protein post‐translational modification, tumour genesis and progression

## Abstract

Cellular lactylation, a recently identified post‐translational modification, has emerged as a crucial regulator in various biological processes, particularly in cancer. The discovery of lactylation provides a new perspective for understanding the functional significance of the Warburg effect in tumour cells. Enzymes involved in the glycolytic pathway modulate lactylation, influencing tumour genesis and progression. This review explores the intricate relationship between glycolysis, lactylation and tumour biology, with a focus on how enzymes participating in glycolysis impact lactylation in cancer cells. We discuss how glycolytic enzymes regulate lactylation and highlight their broader implications in tumour biology. The role of lactylation in shaping the tumour microenvironment underscores its increasing significance as a biomarker for cancer prognosis and a target for therapeutic intervention. Therefore, we also summarised the potential of targeting lactylation as a cancer therapy strategy.

**Key points:**

- Glycolytic enzymes regulate lactylation in cancer cells.
- Lactylation drives tumour growth, metastasis, immune evasion and contributes to microenvironment remodelling
- Targeting lactylation holds promise for cancer therapy.

## Graphical abstract

Graphical Headlights

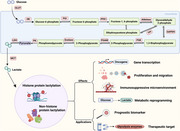

## 1 INTRODUCTION

Cellular metabolism is fundamental for maintaining organismal homeostasis, supporting essential functions such as energy production, biosynthesis and signal transduction.^1^, ^2^ Proper regulation of these metabolic pathways is crucial for sustaining metabolic balance and ensuring normal physiological processes.^3^, ^4^, ^5^ Dysregulation of cellular metabolism is a hallmark of many diseases, especially cancer where metabolic reprogramming is commonly observed. Among these reprogramming events, the reliance of tumour cells on aerobic glycolysis—also known as the Warburg effect—represents a central metabolic adaptation.^6^ This metabolic shift favours glycolytic flux even in the presence of oxygen, resulting in excessive lactate production, a metabolite that profoundly influences both the tumour microenvironment (TME) and intracellular signalling networks.^7^, ^8^

Traditionally regarded as a waste product of anaerobic metabolism, lactate has now been recognised as a signalling molecule and a precursor for post‐translational modifications (PTMs).^9^ One such modification, protein lactylation, involves the covalent attachment of lactyl groups—primarily derived from intracellular lactate—to lysine residues on proteins.^10^, ^11^, ^12^, ^13^ This dynamic and reversible PTM plays crucial roles in gene expression,^14^ chromatin remodelling^15^ and cellular fate decisions,^16^ and accumulating evidence links it to cancer progression.^17^, ^18^ Collectively, these observations suggest that lactate serves as a critical bridge between metabolic reprogramming and epigenetic regulation in tumour cells.

**FIGURE 1:**
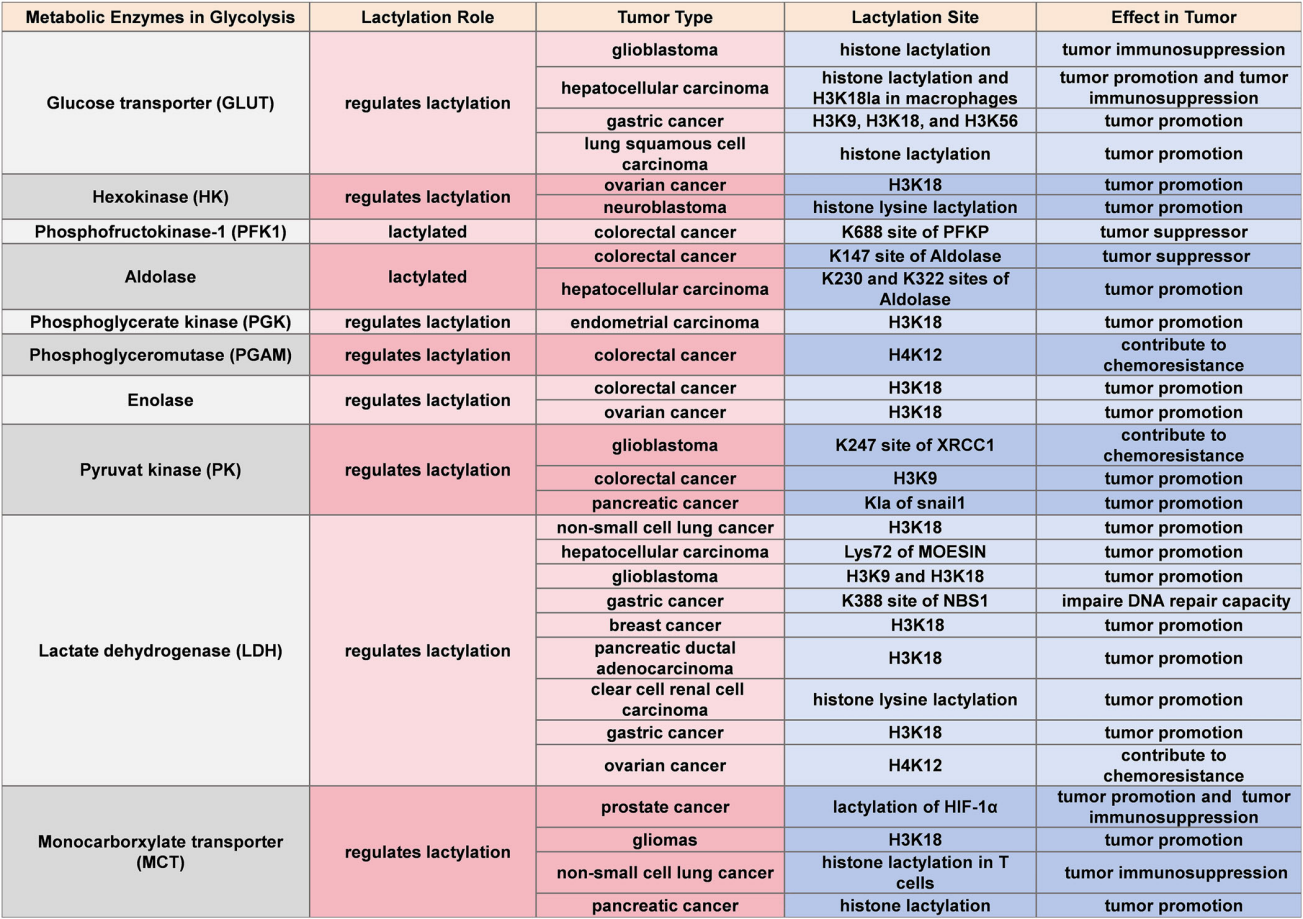
Glycolysis enzymes and lactylation regulation in tumours. Enzymes involved in glycolysis not only function as metabolic regulators but also play key roles in lactylation‐mediated regulation of gene expression, tumour progression and other diseases. These enzymes contribute to disease progression either by promoting gene transcription through histone lactylation or by undergoing lactylation themselves, which modulates their enzymatic activity and influences lactate production. This table categorises glycolytic enzymes based on their involvement in lactylation, distinguishing between enzymes that undergo lactylation and those that regulate lactylation of other proteins, including histones. For lactylated enzymes, specific modification sites (if identified) are listed. The associated tumour types indicate the contexts in which these lactylation‐related modifications have been observed. H3K18, Lysine 18 of histone H3; H3K9, Lysine 9 of histone H3; H3K56, Lysine 56 of histone H3; H4K12, Lysine 12 of histone H4; HIF‐1α, hypoxia‐inducible factor 1‐alpha; K688, Lysine 688; K147, Lysine 147; K230 and K322, Lysine 230 and 322; K247, Lysine 247; K388, Lysine 388; Kla, Lysine lactylation; NBS1, nijmegen breakage syndrome 1; XRCC1, X‐ray cross complementing protein 1.

As the principal producers of lactate through glycolysis, glycolytic enzymes have emerged as key regulators of cellular lactylation dynamics. The key glycolytic enzymes such as hexokinase (HK),^19^ phosphofructokinase‐1 (PFK1)^20^ and lactate dehydrogenase (LDH)^21^ not only drive the metabolic conversion of glucose to lactate, but also regulate lactylation patterns in cancer cells (Figure 1). Given the strong dependence of cancer cells on aerobic glycolysis, the regulatory influence of glycolytic enzymes on protein lactylation is particularly prominent in tumours, where it contributes to altered biological behaviours such as proliferation, immune evasion, metastasis and therapeutic resistance.^22^, ^23^

In this context, lactylation has gained attention as a potential link between metabolic reprogramming and epigenetic remodelling in cancer, offering new perspectives for biomarker development and therapeutic intervention. Thus, understanding the intricate interplay between glycolysis and lactylation in cancer cells is essential for advancing the field of cancer metabolism and identifying novel therapeutic strategies. This review provides a comprehensive analysis of the regulatory roles of glycolytic enzymes in lactylation, examines the impact of lactylation on tumour biology, and explores its potential as a therapeutic target in cancer treatment.

## 2 GLYCOLYSIS AND LACTATE PRODUCTION IN TUMOURS

Glycolysis is an energy‐generating process in eukaryotic cells that occurs in the cytoplasm under hypoxic conditions. Through the action of various enzymes, one glucose molecule is converted into two pyruvate molecules, two nicotinamide adenine dinucleotide (NADH) molecules, two water molecules and two adenosine triphosphate (ATP) molecules.^24^, ^25^ Pyruvate can either enter the mitochondria for oxidative phosphorylation to generate ATP or be metabolised further in the cytoplasm to form lactate.^26^, ^27^ Glycolysis dysregulation is implicated in several diseases. Inflammatory cells, for example, induce glycolysis through the HIF1‐α pathway to create a hypoxic‐like state,^28^ and a similar metabolic shift from oxidative phosphorylation to glycolysis is also observed in neurodegenerative diseases, such as Alzheimer's disease.^29^, ^30^ This phenomenon is more common in tumour cells, which employ aerobic glycolysis even in the presence of sufficient oxygen, known as the ‘Warburg’ effect, which has become an important hallmark of malignancy.^8^, ^31^, ^32^, ^33^ Although this metabolic strategy is less energy efficient, it provides cancer cells with greater adaptive advantages, such as rapidly generating metabolic intermediates to support biosynthesis or modulating the TME. Therefore, glycolysis serves not only as an energy source for cancer cells but also as a critical driver in sustaining their malignant phenotype.

To further sustain these malignant behaviours, cancer cells extensively produce lactic acid, a key metabolic product that plays multifaceted roles in tumour progression. Lactic acid is produced from pyruvate via LDH under hypoxic conditions, which was originally considered solely as a byproduct of glycolysis.^34^, ^35^ However, in the early 20th century, Warburg et al. observed that even in the presence of oxygen, tumour cells preferentially produce lactate through glycolysis, a metabolic shift that promotes tumour growth and migration.^36^, ^37^ Beyond energy production, lactate in cancer cells also supports cellular proliferation, inhibits immune cell function in the TME and promotes tumour angiogenesis.^38^ An article has been reported, lactate serves as an extracellular signalling molecule that binds to the G protein‐coupled receptor 81 (GPR81) on the cell surface, triggering downstream signalling pathways that drive tumour progression^39^ and dampening immune activity.^40^ Excessive lactate production acidifies the TME, inhibiting T‐cell and NK‐cell function, reducing key cytokine secretion (e.g., interferon‐γ), and facilitating immune escape.^41^ Additionally, in senescent cells, upregulation of pyruvate dehydrogenase kinase 4 (PDK4) increases lactate production, contributing to malignancy.^42^ In prostate cancer, lactate promotes intracellular lipid accumulation and acetylation, establishing a metabolic‒epigenetic axis that enhances cancer cell migration.^43^ These findings suggest that lactate not only plays a crucial role in energy metabolism but also directly mediate epigenetic regulation, further influencing tumour progression. Meanwhile, recent study has identified lactylation of lysine 100 in mitochondrial phosphoenolpyruvate carboxykinase 2 (PCK2) during liver transplantation, leading to increased enzymatic activity and exacerbation of ferroptosis in patients.^44^ Furthermore, lactate has been found to play a crucial role in inflammation and fibrosis formation,^45^, ^46^, ^47^ indicating that its impact may extend beyond cancer to a broader range of pathophysiological processes. Therefore, therapeutic strategies targeting glycolysis and lactate metabolism may provide novel insights for the treatment of various diseases.

## 3 OVERVIEW OF LACTYLATION

### 3.1 Lactylation is a novel post‐translational modification of proteins

Beyond serving as a metabolic byproduct, lactate accumulation in tumours has been shown to influence gene regulation through epigenetic pathways. In 2019, Professor Zhao Yingming's team discovered that lactate can induce histone modifications, leading to the upregulation of associated genes and promoting the development of diseases.^48^ This insight has provided new mechanistic understanding of disease progression by linking metabolic abnormalities with heritable regulatory mechanisms.^49^, ^50^ Recent research has further distinguished lactylation on histone proteins from lactylation on non‐histone proteins, both are critically involved in the pathogenesis and progression of diseases. For histones, histone H3 and H4 are the most studied. Histone H3 lysine 18 (H3K18) lactylation, for instance, modulates immune responses by enhancing Arginase 1 (Arg1) expression, promoting macrophage polarisation towards an anti‐inflammatory state.^51^ In myocardial infarction, glycolytic dysregulation and monocarboxylate transporter 1 (MCT1)‐mediated lactate transport occur in the early stages, while lactylation of H3K18 can inhibit inflammatory factors and promote angiogenesis, thus improving cardiac function.^52^ In pancreatic cancer, inhibiting H4K8 and H4K16 lactylation reduces telomerase activity, triggering cancer cell senescence and apoptosis.^53^ Concurrently, research into non‐histone protein lactylation has advanced markedly, revealing its distinct functional roles. In hepatocellular carcinoma (HCC), lactylation of ATP‐binding cassette sub‐family F member 1 (ABCF1)‐K430 correlates with poor prognosis and promotes cancer cell growth and metastasis^54^; in cervical cancer, DCBLD1 (discoidin, CUB and LCCL domain containing 1)‐K172 lactylation stabilises protein expression and activates the pentose phosphate pathway.^55^ Large‐scale lactylome analyses in HCC identified thousands of non‐histone lysine L‐lactylation (Kla) sites, with adenylate kinase 2 (AK2)‐K28 lactylation shown to suppress kinase activity and correlate with adverse outcomes.^18^ In pancreatic ductal adenocarcinoma (PDAC), spindle‐associated protein 1 (NUSAP1) lactylation strengthens its interaction with c‐Myc and HIF‐1α, stabilising LDHA activity and creating a positive feedback loop that accelerates tumour progression.^21^ Overall, histone and non‐histone lactylation complement each other in linking metabolic changes to disease processes, with histone modifications mainly modulating gene expression and non‐histone modifications influencing protein function and signalling.^56^, ^57^

As a PTM, lactylation links cellular metabolism to changes in chromatin structure and transcriptional programs.^58^ Against this backdrop, understanding the broader landscape of epigenetic modifications becomes essential. Such modifications—including DNA methylation and histone modifications—serve as pivotal molecular switches that influence cellular behaviour and contribute to cancer development, as observed in liver and colorectal cancers.^59^, ^60^, ^61^, ^62^, ^63^ Increasing evidence suggests that epigenetic abnormalities often precede genetic mutations and may serve as pivotal events in cancer initiation and progression.^64^ Among the various epigenetic modifications, PTMs are a fundamental regulatory layer. More than 300 distinct PTMs types have been identified to date,^65^ playing a crucial role in cancer development and serving as important cancer markers.^66^ Common PTMs in cancer include methylation, acetylation, phosphorylation, ubiquitination and glycosylation.^67^, ^68^ For example, acetylation of histone H3 at lysine 27 (H3K27ac) promotes the expression of oncogenes, driving cancer cell proliferation and metastasis.^69^, ^70^ Methylation at H3K27 (H3K27me3) is linked to poor chemotherapy outcomes in triple‐negative breast cancer.^71^ These observations suggest that distinct modifications at the same locus may exert opposing functions in cancer, highlighting the complexity and context‐dependent specificity of epigenetic regulation. Beyond histone modifications, PTMs of signalling pathway‐related proteins also play critical roles in tumour initiation and progression. For instance, protein kinase AMP‐activated catalytic subunit α2 (AMPKα2) phosphorylation promotes metastasis and increases mortality.^72^ Ubiquitination and deubiquitination also impact tumour growth and invasiveness^73^, ^74^ with IκBα ubiquitination activation contributing to colorectal cancer development.^75^ Glycosylation regulates cancer signalling and immune processes,^76^, ^77^ and N‐glycosylation of Mer tyrosine kinase (MerTK) at residues 294 and 454 is critical for poor prognosis in liver cancer.^78^ These findings collectively underscore that epigenetic modifications not only regulate cancer cell survival and metastasis but also influence responses to immunotherapy and other treatment modalities.

### 3.2 ‘Writers’, ‘readers’ and ‘erasers’ of lactylation

**FIGURE 2:**
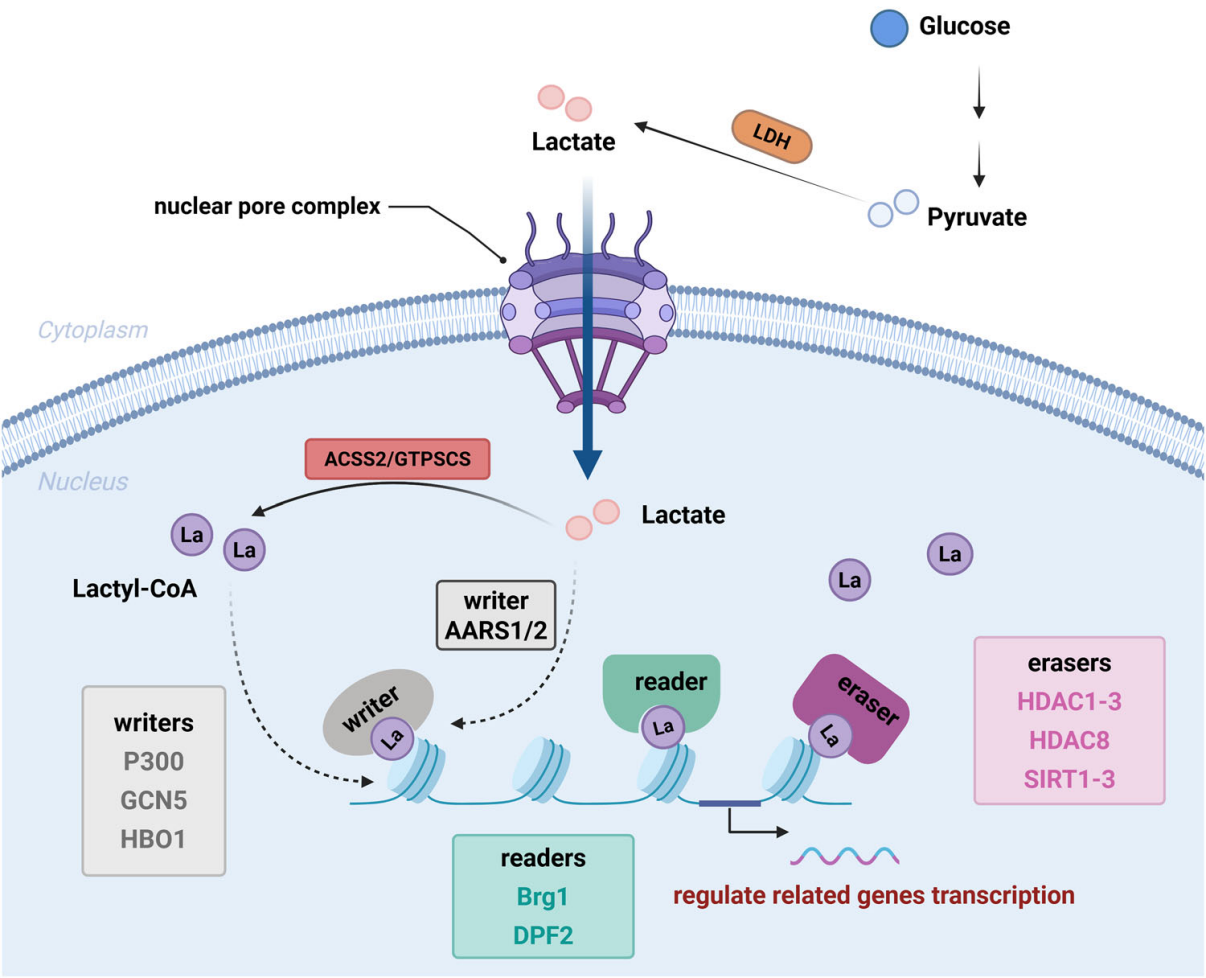
Reading, writing and erasing of lactylation. Upon cellular uptake, a single molecule of glucose undergoes glycolysis to generate two molecules of pyruvate. These pyruvate molecules are subsequently converted into lactate by lactate dehydrogenase (LDH). Under enzymatic regulation, lactate is further processed into lactyl‐CoA, which translocates into the nucleus and participates in lactylation modifications, thereby regulating the expression of target genes. Similar to other post‐translational modifications, lactylation relies on the coordinated actions of ‘writers’, ‘readers’ and ‘erasers’ to establish, recognise and remove the modification, respectively. ‘Writer’ enzymes catalyse histone lactylation by adding lactyl groups to specific lysine residues. Identified writers include P300, GCN5, HBO1 and AARS1/2. P300 was first discovered to regulate histone lactylation in HEK293T cells. Subsequently, GCN5 was identified as a lactyltransferase in monocytes, while HBO1 was found to mediate histone lactylation in lactate‐enriched HEK293T and HeLa cells. Recent evidence suggests that AARS1/2 can directly utilise lactate as a lactyl donor in cellular lactylation. ‘Reader’ proteins recognise and bind to lactylation marks, facilitating interactions with lactylated histones to recruit other factors involved in gene expression regulation and cellular processes. Identified readers include Brg1, which plays a role in pluripotent stem cells, and DPF2, which has been implicated in lactylation‐associated regulation in cervical cancer. ‘Eraser’ enzymes remove histone lactylation marks, restoring histones to their original state. Currently identified erasers include HDAC1‒3, HDAC8 and SIRT1‒3, which have been shown to participate in the regulation of lactylation in HEK293T and HeLa cells in vitro assays. AARS1/2, alanyl‐tRNA synthase 1/2; Brg1, brahma‐related gene 1; DPF2, double plant homeodomain finger 2; GCN5, general control nonderepressible 5; HBO1, histone acetyltransferase binding to ORC1; HDAC, histone deacetylase; P300, E1A‐binding protein p300; SIRT, sirtuins.

Similar to other PTMs, lactylation also involves the coordinated action of ‘writers’, ‘erasers’ and ‘readers’ (Figure 2). These elements fine‐tune the chemical state of histones to control chromatin accessibility and transcriptional programs, thereby influencing cellular functions and fate.^9^

#### 3.2.1 Writers

In 2019, p300 was the first identified lactylation ‘writer’, and research has shown that its overexpression in HEK293T cells slightly increases histone Kla levels.^48^ Subsequently, a study on myocardial infarction revealed that general control nonderepressible 5 (GCN5) catalyses the lactylation of H3K18.^52^ Later, a 2024 study identified that the lysine lactyltransferase histone acetyltransferase binding to ORC1 (HBO1) can regulate histone Kla both in vitro and in cells, potentially preferentially catalysing H3K9la.^79^ Meanwhile, lysine acetyltransferase 5 (KAT5, also known as tat interacting protein 60 kDa [TIP60]) has been found to promote the lactylation of the non‐histone protein nijmegen breakage syndrome 1 (NBS1) at lysine 388 in cancer cells, indicating its function as a lactyltransferase.^80^ In 2025, research demonstrated that Acyl‐CoA synthetase short chain family member 2 (ACSS2), in conjunction with lysine acetyltransferase 2A (KATA2)/LDHA, functions as a metastatic enzyme in breast cancer, regulating the expression of related genes.^81^ Furthermore, recent findings suggest that alanyl‐tRNA synthase 1/2 (AARS1/2) can catalyse lactylation without the involvement of coenzyme lactate.^82^

#### 3.2.2 Erasers

Research on lactylation ‘erasers’ has identified two principal classes of enzymes: class I histone deacetylases (HDACs) and sirtuins (SIRTs). In 2022, class I HDACs (HDAC1–3) were first reported to exhibit delactylase activity, confirming that lactylation can be dynamically reversed by specific enzymes.^83^, ^84^ Subsequently, members of the sirtuin family were also found to possess delactylase functions. SIRT2 was shown to reduce histone lactylation levels in neuroblastoma, thereby suppressing cell proliferation and migration.^85^ SIRT3 displayed high delactylase activity towards H4K16la, effectively removing this modification.^86^ In addition, both SIRT1 and SIRT3 have been implicated in the removal of lactyl groups from histone and non‐histone proteins; notably, delactylation of PKM2 at lysine 207 by these enzymes preserves its ATP‐binding capacity and contributes to the maintenance of lactylation homeostasis in mammalian cells.^87^

#### 3.2.3 Readers

Research on lactylation ‘readers’ is relatively limited, a study on induced pluripotent stem cell (iPSC) reprogramming published in 2024 revealed that brahma‐related gene 1 (Brg1) functions as a lactylation reader.^88^ Later that year, another research team discovered that double plant homeodomain finger 2 (DPF2) can bind to H3K14la, facilitating transcriptional activation and contributing to cervical cancer development.^89^ Moreover, a non‐enzymatic form of lactylation modification was identified. In tissues with elevated glycolysis, S‐D‐lactoylglutathione has been shown to transfer lactate groups to lysine residues on non‐histone proteins, thereby effectuating the modification.^90^

‘Writers’, ‘erasers’ and ‘readers’ play central roles in regulating lactylation and form an interdependent, dynamic network that fine‐tunes lactylation in response to metabolic and signalling cues. Acetyltransferases such as p300 (acting as writers) and deacetylases including HDAC family members (acting as erasers) not only execute their individual modifications but also interact in a cooperative or antagonistic manner, potentially determining the balance between lactylation and other PTMs. For example, lactylation can upregulate alpha‐ketoglutarate‐dependent dioxygenase homologue 3 (ALKBH3), which in turn promotes N1‐methyladenosine (m1A) demethylation of SP100A, facilitating cancer cell invasion.^17^ In hepatic stellate cell (HSC) activation, H3K18 lactylation and acetylation appear to compete at the same site, with lactylation dominance promoting liver fibrosis.^19^ Recently, both in vivo and in vitro studies have shown that HDAC1–3‐mediated delactylation is reversible and dependent on intracellular lactate levels.^91^ This context‐dependent reversibility, contrasting with the canonical eraser function of HDACs, underscores the dynamic balance between writers and erasers in regulating lactylation. Furthermore, the expression and activity of these regulators are highly context dependent. Tumour cells, infiltrating immune cells and stromal components display distinct patterns of lactylation‐related enzyme expression, resulting in diverse functional outcomes. In colorectal cancer, p300‐mediated H3K18 lactylation enhances polymorphonuclear myeloid‐derived suppressor cell (PMN‐MDSC) infiltration and promotes immunosuppression,^92^ whereas in macrophages, p300‐catalysed histone lactylation facilitates M2 polarisation and inflammation resolution.^48^ These examples illustrate the context‐dependent roles of p300 across immune and TMEs. Therefore, systematic analyses across multiple cell types and microenvironmental contexts are essential to fully elucidate lactylation dynamics in cancer, and further mechanistic and translational studies are warranted.

## 4 GLYCOLYSIS ENZYMES PLAY IMPORTANT ROLE IN LACTYLATION OF CANCER

**FIGURE 3:**
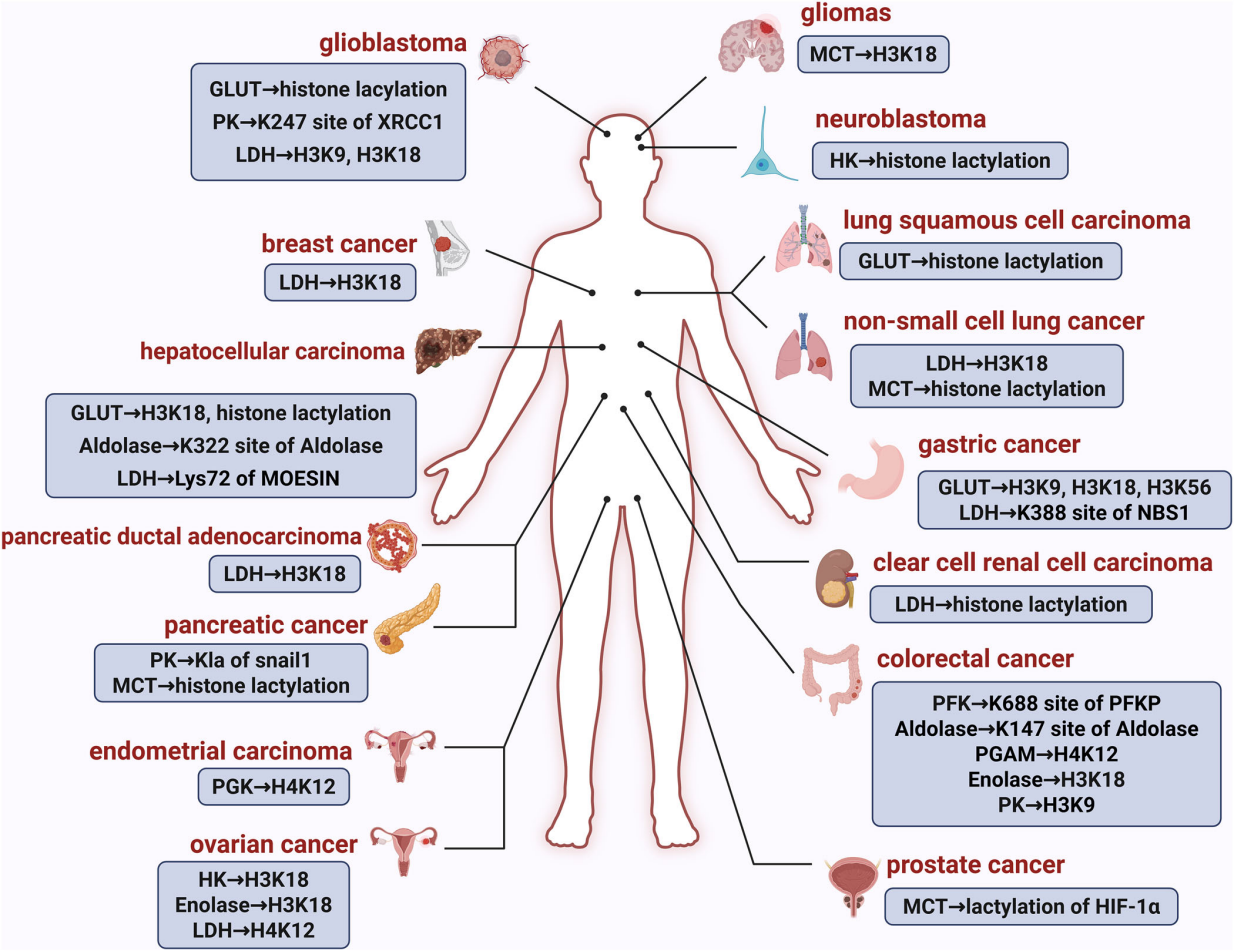
Research summary of lactylation regulated by glycolysis across cancers. Human cancers are illustrated with key glycolytic enzymes that either undergo lactylation or regulate lactylation on other proteins, including histones. This diagram provides a visual overview of the connections between glycolysis, lactylation and tumour.

Building upon the preceding discussion, glycolysis not only supports the metabolic demands of cancer cells but also contributes to the regulation of intracellular lactate levels through the activity of glycolytic enzymes.^33^, ^93^, ^94^ In light of the reliance of tumour cells on aerobic glycolysis, it is plausible that glycolytic enzymes function as key regulators of lactylation dynamics, with especially significant roles in cancer.^95^ In this context, the regulatory functions of glycolytic enzymes and lactylation within cancer cells deserves detailed exploration (Figure 3).

### 4.1 Glucose transporter

Glucose transporter (GLUT), a family of 14 distinct isoforms with specialised functions, facilitate the uptake of extracellular glucose into the cytoplasm for cellular metabolism.^96^ Given the strong association between dysregulated glucose metabolism and cancer, GLUTs play a critical role in tumour progression.^32^, ^97^ Emerging evidence suggests that GLUT‐mediated glucose transport is intricately linked to histone lactylation, thereby influencing tumour development and immune regulation. For instance, in glioblastoma (GBM), protein kinase R‐like endoplasmic reticulum kinase (PERK) drives GLUT1 overexpression, leading to increased glycolysis and intracellular histone lactylation, which promotes IL‐10 secretion and establishes an immunosuppressive microenvironment.^98^ In HCC, serine/arginine‐rich splicing factor (SRSF)‐mediated upregulation of GLUT1 and glycolytic enzymes induces histone lactylation and forming a positive feedback loop that sustains tumour progression. The accumulated lactate is further transported into macrophages, triggering H3K18 lactylation and promoting M2 polarisation, thereby reinforcing immune tolerance.^99^ Beyond this modulation, GLUTs also exert direct effects on tumour growth and invasion through histone lactylation. In gastric cancer, GLUT3 upregulation enhances histone lactylation at H3K9, H3K18 and H3K56, accelerating cancer cell proliferation and migration, which is associated with poor prognosis.^100^ Moreover, research in lung squamous cell carcinoma have shown that solute carrier family 2 member (SLC2A1) upregulation increases GLUT1 expression and histone lactylation, although its precise role in tumour regulation remains unclear.^101^ These findings highlight the interplay between GLUTs, histone lactylation and tumour progression, although their precise roles remain to be elucidated.

### 4.2 Hexokinase

HK is the first rate‐limiting enzyme in the glycolytic pathway, responsible for phosphorylating glucose to form glucose‐6‐phosphate, thereby initiating the glycolytic pathway.^32^ HKs have four distinct isoforms: HK1, HK2, HK3 and HK4, which have been found to regulate the initiation and progression of various cancers through both metabolic and non‐metabolic functions.^102^, ^103^, ^104^ Recent studies highlight that HKs not only play a key role in glycolysis but also modulate cellular programs via histone lactylation, thereby participating in multiple cellular functions and pathological processes. For instance, in neuroblastoma, HK3 has been found to promote the intracellular histone lactylation levels and drive macrophage polarisation towards the M2 phenotype, thereby contributing to tumour progression, including proliferation, migration and invasion.^105^ This finding suggests that HK3 may function not only as a glycolytic enzyme but also as a regulator of the TME by modulating immune cell states. In ovarian cancer cells, Tanshinone I treatment inhibits the expression of HK2, reducing lactate production and consequently lowering the lactylation level of histone H3K18, which in turn suppresses the expression of multiple genes, including threonine tyrosine kinase (TTK), platelet derived growth factor receptor beta (PDGFRβ), YTH N6‐methyladenosine RNA‐binding protein 2 (YTHDF2) and rubicon like autophagy enhancer (RUBCNL).^106^ Notably, the similar regulatory role of HK2 is not limited to cancer. In microglial cells, BTB domain and CNC homologue 1 (BACH1) reduces HK2 expression, thereby decreasing lactate accumulation and lowering the lactylation level of H4K12, which affects the expression of leucine‐rich repeat containing 15 (LRRC15) and regulates the interaction between microglia and astrocyte precursors.^107^ In HSCs, the expression and lactylation of HK2 influence cell activation and liver fibrosis.^19^ Moreover, H3K18 lactylation can further upregulate HK2 expression, forming a positive feedback loop that helps cells maintain high glycolytic activity under stress conditions.^108^ This mechanism plays a significant role in various pathological states, suggesting that glycolysis and lactylation may jointly establish a metabolism‒epigenetics regulatory axis, enabling cells to adapt to various environmental stresses.

### 4.3 Phosphofructokinase‐1

PFK1 is the second rate‐limiting enzyme in glycolysis, with three isoforms: PFKL, PFKP and PFKM. It catalyses the conversion of fructose‐6‐phosphate to fructose‐1,6‐bisphosphate (FBP).^109^, ^110^, ^111^ Studies have shown that the lactylation level of PFKM significantly decreases in normal adult tissues, whereas after myocardial infarction, the lactylation level of PFKM significantly increases. This suggests that lactylation modifications near the catalytic site of PFKM can regulate its catalytic activity and glycolytic rate, thereby promoting cardiac regeneration.^112^ This study provides new therapeutic insights for cardiovascular disease research. Nuclear receptor subfamily 4 group A member 3 (NR4A3) has been shown to bind to the promoter regions of PFKL and aldolase, enhancing glycolysis and subsequently increasing lactate production. The resulting accumulation of lactate elevates H3K18 lactylation, upregulating Phospho1 expression and contributing to arterial calcification.^113^

Although PFK1 has not yet been shown to directly regulate the lactylation process in tumours, growing evidence suggests that PFK1 itself can undergo lactylation, thereby influencing tumour progression. In colon cancer, the decreased lactylation level of non‐histone PFKP enhances its catalytic activity and promotes glycolysis, while lactate and PFKP lactylation establish a negative feedback mechanism that regulates glycolysis in normal tissues.^20^ This discovery proposes a potential dynamic equilibrium between glycolysis and lactylation, meriting further investigation across various tumour types, particularly to determine whether this negative feedback mechanism contributes to metabolic homeostasis in different tissues.

### 4.4 Aldolase

Aldolase exists in three isoforms: Aldolase A, B and C, which are distributed in different tissues of the human body.^114^ It catalyses the reversible conversion of FBP to glyceraldehyde 3‐phosphate (GAP) in the glycolytic pathway.^115^, ^116^ Aldolase has been found to be upregulated in various cancers, where it plays a role in regulating cancer cell proliferation and migration.^117^, ^118^ Despite the current lack of direct evidence linking Aldolase to the modulation of lactylation in tumours, emerging research suggests that Aldolase may indirectly participate in regulating lactylation processes. For example, Aldolase B activates downstream effector molecules, such as the CEA cell adhesion molecule‐6 (CEACAM6), to promote histone lactylation and contribute to tumour biology.^119^ Furthermore, during liver fibrosis, Aldolase A is significantly upregulated, generating large amounts of lactate, which drives the accumulation of H3K18 lactylation and modulates cellular programs relevant to disease progression.^120^, ^121^

In the context of tumours, Aldolase has been found to undergo lactylation, which in turn modulates its enzymatic activity and influences cancer cell behaviour. In liver cancer stem cells (LSCS), the cell proliferation capacity and glycolytic activity are significantly associated with lactylation at Aldolase A lysine residues K230/K322 and histone3 lysine residue K56. This lactylation promotes histone lactylation through a positive feedback loop, maintaining stem cell properties and enhancing liver carcinogenesis.^122^ Moreover, an analysis based on genetic code expansion (GCE) demonstrated that lactylation at ALDOA‐K147 reshapes its interaction with FBP, leading to reduced enzymatic activity, enhanced stability and altered subcellular distribution, indicating its potential as a therapeutic target in cancer.^123^

### 4.5 Phosphoglycerate kinase

Phosphoglycerate kinase (PGK) exists in two isoforms: PGK1 and PGK2, with PGK2 being expressed only during spermatogenesis, while PGK1 is present in most cells.^124^ In the sixth step of glycolysis, PGK1 catalyses the reversible conversion of 1,3‐bisphosphoglycerate to 3‐phosphoglycerate (3‐PG), simultaneously generating ATP.^125^ Beyond its canonical metabolic role, PGK1 also functions as a protein kinase, phosphorylating substrates such as Beclin1 and pyruvate dehydrogenase kinase 1 (PDHK1), thereby contributing to tumourigenesis.^126^ It also functions as a transcriptional coactivator to regulate β‐catenin, affecting tumour proliferation and migration.^127^ These non‐metabolic functions suggest that PGK1 may serve as a bridge between metabolism and signalling, playing a crucial role in adaptive changes in cancer cells. Furthermore, PGK1 influences tumour progression through PTMs such as acetylation,^128^ phosphorylation,^129^ ubiquitination^130^ and glycosylation.^131^ Recent studies have shown^132^ that in endometrial cancer, the lactylation level of histone H3K18 is significantly increased, directly regulating the expression of ubiquitin‐specific peptidase 39 (USP39). USP39, by deubiquitinating PGK1, stabilises PGK1 and activates the PI3K/AKT/HIF‐1α signalling pathway, promoting glycolysis. This forms a positive feedback loop that plays an important role in the malignant progression of tumours, suggesting that lactylation extends beyond histone modifications to regulate cancer metabolic adaptation by targeting key enzymes such as PGK1. However, whether PGK1 undergoes lactylation and its impact on enzymatic activity and signalling remain unclear. Further investigation is needed to elucidate potential crosstalk between lactylation and other PTMs.

### 4.6 Phosphoglycerate mutase

Phosphoglycerate mutase (PGAM) catalyses the interconversion between 2‐phosphoglycerate (2‐PG) and 3‐PG. In mammals, there are two isoforms: PGAM1 and PGAM2,^133^ with PGAM1 playing a critical role in cancer aerobic glycolysis and being significantly associated with poor prognosis in cancer patients.^134^, ^135^ Research has found that PGAM1 has a significant inhibitory effect on tumour immunity. In breast cancer cells, it regulates C‒C motif chemokine ligand 2 (CCL2) expression, promoting macrophage recruitment and M2 polarisation, thereby inducing an immunosuppressive TME and driving tumour progression.^136^ Overexpression of PGAM1 in cancer cells impairs the memory and antitumour function of CD8+ T cells,^137^ whereas inhibition of PGAM1 expression in HCC enhances CD8+ T‐cell infiltration,^138^ suggesting its potential as a therapeutic target. However, PGAM1 functions vary across cancer types. A study^139^ has shown that in colorectal cancer, PGAM1 activity is significantly reduced, leading to the accumulation of 3‐PG and increased lactate production. Lactate, through histone lactylation modifications, regulates drug efflux pathways, including ABC transporter family members, thereby decreasing cellular sensitivity to chemotherapy. In summary, the direct relationship between PGAM and lactylation remains complex and requires further investigation.

### 4.7 Enolase

Enolase exists in three isoforms in mammals: Eno1, Eno2 and Eno3.^140^ It catalyses the reversible conversion of 2‐PG to phosphoenolpyruvate (PEP)^141^ and plays a crucial role in promoting cancer cell survival, proliferation^142^, ^143^ and invasiveness.^144^ For example, in HCC, enolase 1 (ENO1), as an RNA‐binding protein, recruits CCR4‐NOT transcription complex subunit 6 (CNOT6) to promote the mRNA decay of iron regulatory protein 1 (IRP1) in cancer cells, thus inhibiting the expression of Mitoferrin‐1 (Mfrn1) and suppressing mitochondrial iron‐dependent ferroptosis.^145^ Functionally pivotal in aerobic glycolysis, enolase upregulation promotes metabolic reprogramming and lactate production in cancer cells.^146^, ^147^, ^148^ This phenomenon underscores the central role of glycolysis in the energy metabolism of cancer cells. The accumulation of lactate is not merely a byproduct of metabolism, which is also alters epigenetic modifications. Research has shown that in colorectal cancer, the accumulation of lactate leads to the lactylation of H3K18, which activates the transcription of the NOP2/Sun RNA methyltransferase 2 (NSUN2). Lactate also directly modifies the Lys356 residue of NSUN2, enhancing its binding ability to m5C‐modified RNA, further promoting the expression of ENO1. This creates a positive feedback loop, facilitating cancer progression.^149^ Furthermore, Jin et al.^106^ found that Tanshinone I can inhibit the expression of glycolytic enzymes, including enolase, reducing H3K18 lactylation and suppressing the expression and translation of related oncogenes. These studies suggest that Enolase could serve as an effective therapeutic target.

### 4.8 Pyruvate kinase

Pyruvate kinase (PK) is the third rate‐limiting enzyme in glycolysis, catalysing the conversion of PEP to pyruvate, while generating ATP.^150^ PK exists in four isoforms: PKL, PKR, PKM1 and PKM2,^151^ with PKM2 being crucial for aerobic glycolysis in cancer cells.^152^, ^153^ As a key metabolic regulator, lactate not only shapes the TME but also influences cellular transcription through lactylation, further driving cancer progression. For instance, in GBM, the interaction between PKM2 and aldehyde dehydrogenase 1 family member A3 (ALDH1A3) enhances PKM2 tetramerisation, promoting lactate accumulation and subsequent lactylation of X‐ray repair cross complementing gene 1 (XRCC1) at K247. This modification alters the protein surface from negatively charged to neutral, facilitating its binding to importin α, thereby enhancing DNA repair capability and increases resistance to radiotherapy and chemotherapy.^154^ In colorectal cancer, methyltransferase 1 (METTL1) mediates the m7G methylation of PKM mRNA, promoting the expression of PKM2, which enhances lactate production, induces H3K9 lactylation, and further activates METTL1 transcription. This forms a positive feedback loop, accelerating tumour progression.^155^ This mechanism illustrates the integration of metabolic and regulatory networks, providing potential targets for therapeutic intervention in tumour metabolism. In pancreatic cancer, the transcription of PKM2 is activated by c‐Myc, promoting glycolysis and lactate production, which leads to lactylation of Snail1 and ultimately facilitates epithelial‒mesenchymal transition (EMT) and tumour growth.^156^ Additionally, the upregulation of PKM2‐mediated histone lactylation plays an important role in various conditions, including kidney fibrosis,^157^ flap fibrosis,^158^ alleviation of osteoporosis^159^ and cochlear development.^160^ In conclusion, the relationship between PK and lactylation has gained increasing attention, highlighting the need for further investigation into their intricate regulatory mechanisms.

### 4.9 Lactate dehydrogenase

LDH catalyses the final step of aerobic glycolysis, converting pyruvate into lactate, and is one of the key enzymes driving the Warburg effect in tumours.^161^, ^162^ Comprising two isoforms, LDHA and LDHB, both have been implicated in cancer progression and hold significant clinical relevance.^163^, ^164^, ^165^ Recent evidence also underscores its role in regulating histone lactylation, linking metabolic activity to epigenetic modulation. The influence of LDH on histone lactylation and tumour progression is increasingly evident. Elevated LDHA activity has been associated with increased H3K18 lactylation, a modification that promotes immune escape and correlates with poor prognosis in non‐small cell lung cancer (NSCLC).^166^ Similarly, in HCC, lactate promotes the lactylation of MOESIN at Lys72, which forms additional hydrogen bonds with transforming growth factor‐β receptor I (TGF‐βRI), thereby enhancing transforming growth factor‐β (TGF‐β) signalling. Notably, combined PD‐1 and LDH inhibitor (LDHi) therapy exhibits superior efficacy compared to PD‐1 blockade alone.^167^ In GBM, inhibition of LDHA/B suppresses H3K9 and H3K18 lactylation, enhancing tumour sensitivity to temozolomide (TMZ) and CAR‐T therapy.^168^, ^169^ While inhibition of LDHA reduces NBS1 K388 lactylation, this modification alters the protein's conformation, disrupting its interaction with meiotic recombination 1‐Rad50 (MRE11‐RAD50) to form the MRN complex and impairing DNA repair capacity.^80^ Beyond immune modulation, LDH‐mediated lactylation provides a mechanistic link between glycolytic metabolism and tumour cell regulatory networks. In breast cancer, potassium two‐pore domain channel subfamily K member 1 (KCNK1) interacts with LDHA to enhance lactate production, leading to increased H3K18 lactylation and promoting cancer cell proliferation and migration.^170^ Meanwhile, phosphorylation of LDHA by TTK protein kinase in PDAC strengthens its enzymatic activity, further promoting H3K18 lactylation and tumour invasion.^171^ A similar mechanism is observed in clear cell renal cell carcinoma (ccRCC), where LDHA phosphorylation by FKBP prolyl isomerase 10 (FKBP10) accelerates lactate accumulation and histone lactylation, exacerbating tumour progression.^172^ In gastric cancer, a feedback loop involving lncRNA H19, glycolysis and H3K18 lactylation has been identified, with LDHA inhibition reducing lactylation levels and offering a potential therapeutic strategy.^173^ Additionally, lactylation at H4K12, driven by upregulated glycolytic enzymes including LDHA and PKM and contributes to chemoresistance in ovarian cancer.^174^ Beyond transcriptional regulation, LDHA‐induced lactylation also modulates autophagy, further influencing tumour progression.^175^, ^176^ Given its extensive role in tumour progression and therapy resistance, targeting LDH presents a promising strategy for disrupting lactate‐driven oncogenic pathways.

### 4.10 Monocarboxylate transporter

Monocarboxylate transporters are a family of 14 transmembrane proteins, among which MCT1 and MCT4 are involved in lactate transport and play crucial roles in tumour metabolism.^177^, ^178^, ^179^ The impact of MCT1‐mediated lactate transport extends beyond metabolic adaptation, influencing histone lactylation and downstream oncogenic pathways. In prostate cancer, lactate imported via MCT1 facilitates the lactylation of HIF‐1α, stabilising its activity under normoxic conditions and promoting cell migration‐inducing protein (KIAA1199) transcription to drive angiogenesis.^180^ At the same time, MCT4‐mediated lactate export reinforces this effect by inducing HIF‐1α lactylation, which enhances PD‐1 transcription while suppressing Sema3A, ultimately fostering an immunosuppressive TME.^181^ A similar mechanism is evident in gliomas, where MCT1 upregulation elevates lactate levels, leading to increased H3K18 lactylation and TNF superfamily member 9 (TNFSF9) expression, which in turn skews macrophages towards an M2‐like phenotype, thereby accelerating tumour progression.^182^ In addition to its metabolic role in solid tumours, MCT1 dysregulation modulates lactate transport and the TME, providing a basis for exploring its impact on tumour biology. In NSCLC, excessive lactate influx through MCT1 drives histone lactylation in T cells, resulting in heightened PD‐1 expression and impaired antitumour immunity.^183^ Similarly, in malignant pleural effusion (MPE), FOXP3+ NKT cells exhibit high MCT1 expression, which increases intracellular lactylation and dampens their cytotoxic function.^184^ Meanwhile, in pancreatic cancer, upregulation of *SLC16A1*, the gene encoding MCT1, facilitates lactate accumulation and histone lactylation, correlating with enhanced tumour proliferation, migration and poor prognosis.^35^ Notably, the influence of MCT1 is not confined to cancer; its ability to elevate intracellular lactate and induce H3K18 lactylation has also been implicated in idiopathic pulmonary fibrosis, suggesting broader pathological relevance.^185^ These findings collectively emphasise the pivotal role of MCT1 in mediating lactate‐driven pathological processes across diverse diseases. The development of AZD3965, a selective MCT1 inhibitor currently in clinical trials, provides proof‐of‐concept for targeting lactate transport to simultaneously impair tumour metabolism and modulate lactylation.^186^ Thus, MCT1 is a promising avenue for therapeutic intervention.

### 4.11 Potential links between other glycolytic enzymes and lactylation

Phosphoglucose isomerase (PGI), triosephosphate isomerase (TPI) and glyceraldehyde 3‐phosphate dehydrogenase (GAPDH) are important glycolytic enzymes implicated in cancer metabolism; however, their roles in histone lactylation remain undefined. PGI catalyses the interconversion of glucose‐6‐phosphate and fructose‐6‐phosphate^187^, ^188^ and is closely associated with tumour invasiveness.^189^, ^190^, ^191^ Its downregulation suppresses tumour growth^192^ while its overexpression promotes EMT in breast cancer.^193^ TPI, which facilitates the reversible conversion of dihydroxyacetone phosphate (DHAP) and glyceraldehyde 3‐phosphate (GAP),^194^ is frequently upregulated in tumours through PTMs^148^ and regulatory factors such as METTL5, contributing to metabolic reprogramming and tumour progression.^195^, ^196^, ^197^ GAPDH, beyond its role in catalysing the conversion of GAP to 1,3‐bisphosphoglycerate,^198^ is highly dysregulated in cancer and implicated in non‐metabolic processes such as mRNA regulation, autophagy and disease progression.^199^, ^200^, ^201^, ^202^, ^203^ While recent studies suggest a link between GAPDH activity and lactylation in microglial cells,^107^ direct evidence supporting the involvement of GAPDH, PGI or TPI in histone lactylation within the context of cancer remains limited.

Cancer progression is a multifactorial process that encompasses diverse biological activities such as proliferation, migration and metabolic adaptation. In this context, glycolysis‐related enzymes and transporters exert distinct yet interconnected influences on tumour development through lactylation‐dependent mechanisms. As summarised in this section, GLUTs, MCTs and other metabolite carriers primarily facilitate tumour proliferation and metabolic flexibility by modulating glucose and lactate fluxes. Intermediate glycolytic enzymes, including HK, PFK, aldolases, GAPDH and PGK, function as central metabolic hubs that bridge glycolytic flux with epigenetic remodelling and transcriptional reprogramming. Through lactylation‐mediated gene regulation, these enzymes coordinate cellular programs that drive tumour proliferation and migration. Downstream enzymes, such as enolase, PKM2 and LDHA, largely determine lactate output and are closely associated with tumour migration, invasion and immune modulation, underscoring the integration of lactate metabolism with oncogenic signalling pathways.

Together, these glycolytic regulators constitute a hierarchically organised and dynamic network through which glycolytic activity continuously shapes lactate‐dependent tumour phenotypes, providing new insights into how metabolic and lactylation‐driven epigenetic mechanisms jointly influence cancer progression and therapy response.

## 5 BIOLOGICAL FUNCTIONS AND THERAPEUTIC TARGETING OF LACTYLATION IN CANCER

**FIGURE 4:**
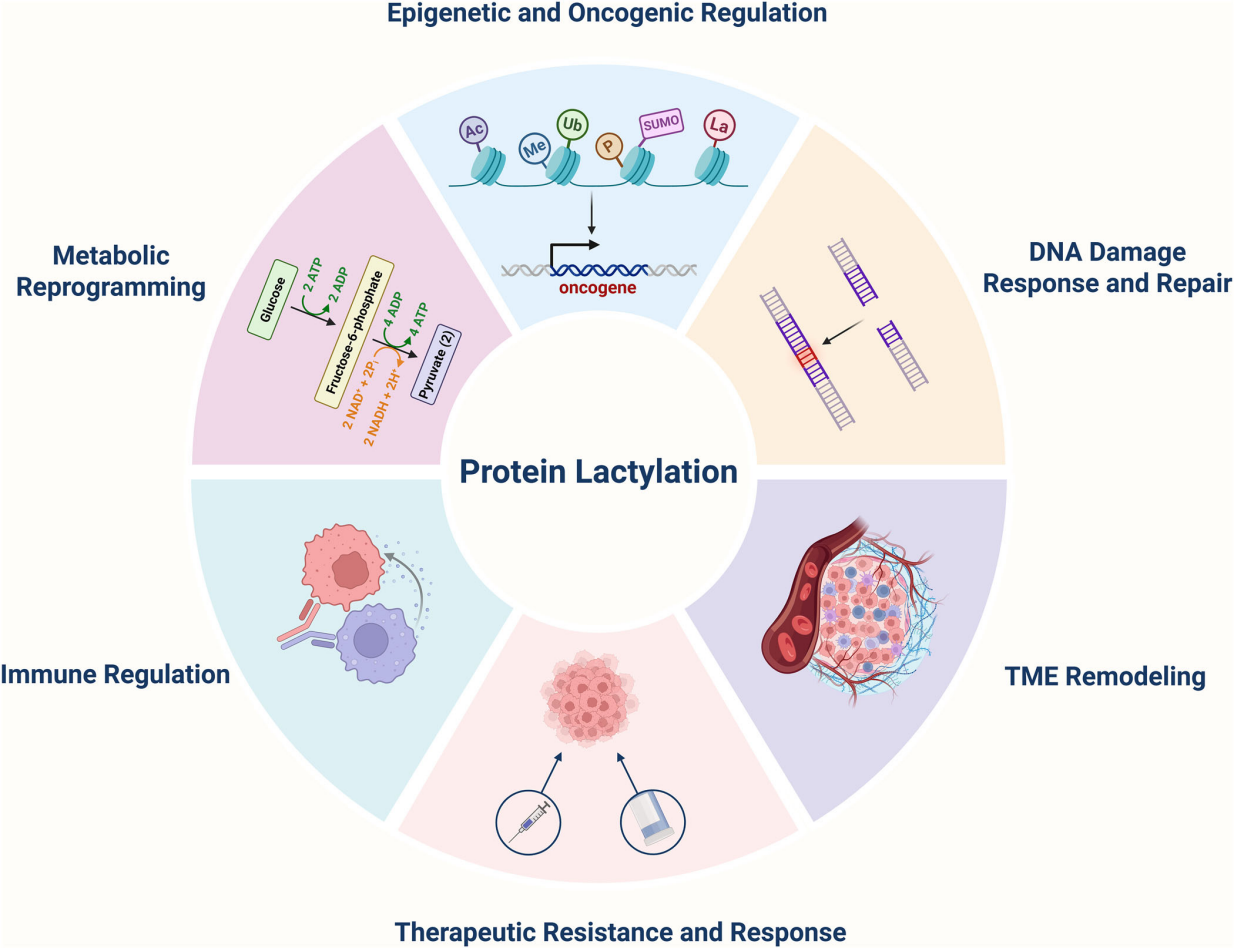
Biological functions of protein lactylation during tumour progression. As a post‐translational modification (PTM), protein lactylation in cancer biology can be categorised into six major functional modules: (1) metabolic reprogramming, achieved through the regulation of glycolytic enzymes and lactate‐driven feedback loops; (2) immune regulation, including M2 macrophage polarisation, CD8⁺ T‐cell exhaustion, and the formation of an immunosuppressive microenvironment; (3) therapeutic resistance and response, whereby lactylation inhibits ferroptosis and promotes metabolic adaptation, thus reducing the efficacy of chemotherapy and immunotherapy; (4) tumour microenvironment (TME) remodelling, exemplified by lactate exchange among tumour cells, cancer‐associated fibroblasts (CAFs) and tumour‐associated Schwann cells (TASCs); (5) DNA damage response and repair, as lactylation helps restore homologous recombination repair pathways; and (6) epigenetic and oncogenic regulation, involving the activation of oncogenic transcription factors such as MCM7 and the maintenance of cancer cell traits through crosstalk with other PTMs.

As previously discussed, lactylation, as a form of PTM, exerts multifaceted biological functions during tumour progression (Figure 4). It not only modulates tumour metabolic reprogramming by regulating the activities of glycolysis‐related enzymes,^204^ but also orchestrates functional outcomes in tumour cells and extends to diverse components of the TME,^205^ thereby influencing both tumour‐intrinsic and extrinsic processes. For instance, in diabetic PDAC, tumour‐associated Schwann cells (TASCs) promote METTL16‐K269 lactylation through exogenous lactate uptake, impairing effector T‐cell function, while K63 lactylation of endosulfine α activates downstream signalling to facilitate tumour‐associated macrophage (TAM) accumulation, with both mechanisms contributing to the establishment of an immunosuppressive microenvironment.^206^, ^207^ Lactate accumulation in the TME further drives M2 macrophage polarisation and CD8⁺ T‐cell exhaustion, reducing immunotherapy efficacy.^99^, ^208^ In stromal elements such as cancer‐associated fibroblasts (CAFs), glycolysis‐derived lactate induces histone lactylation, enhancing glycolysis via a positive feedback loop and contributing to therapeutic resistance.^209^ Additionally, lactate from perineural invasion‐associated CAFs (pCAFs) can be taken up by tumour cells, elevating H3K18la levels and promoting tumour progression.^210^ Collectively, these observations underscore lactylation as a central regulator of TME remodelling, immune suppression and therapy resistance.

**FIGURE 5:**
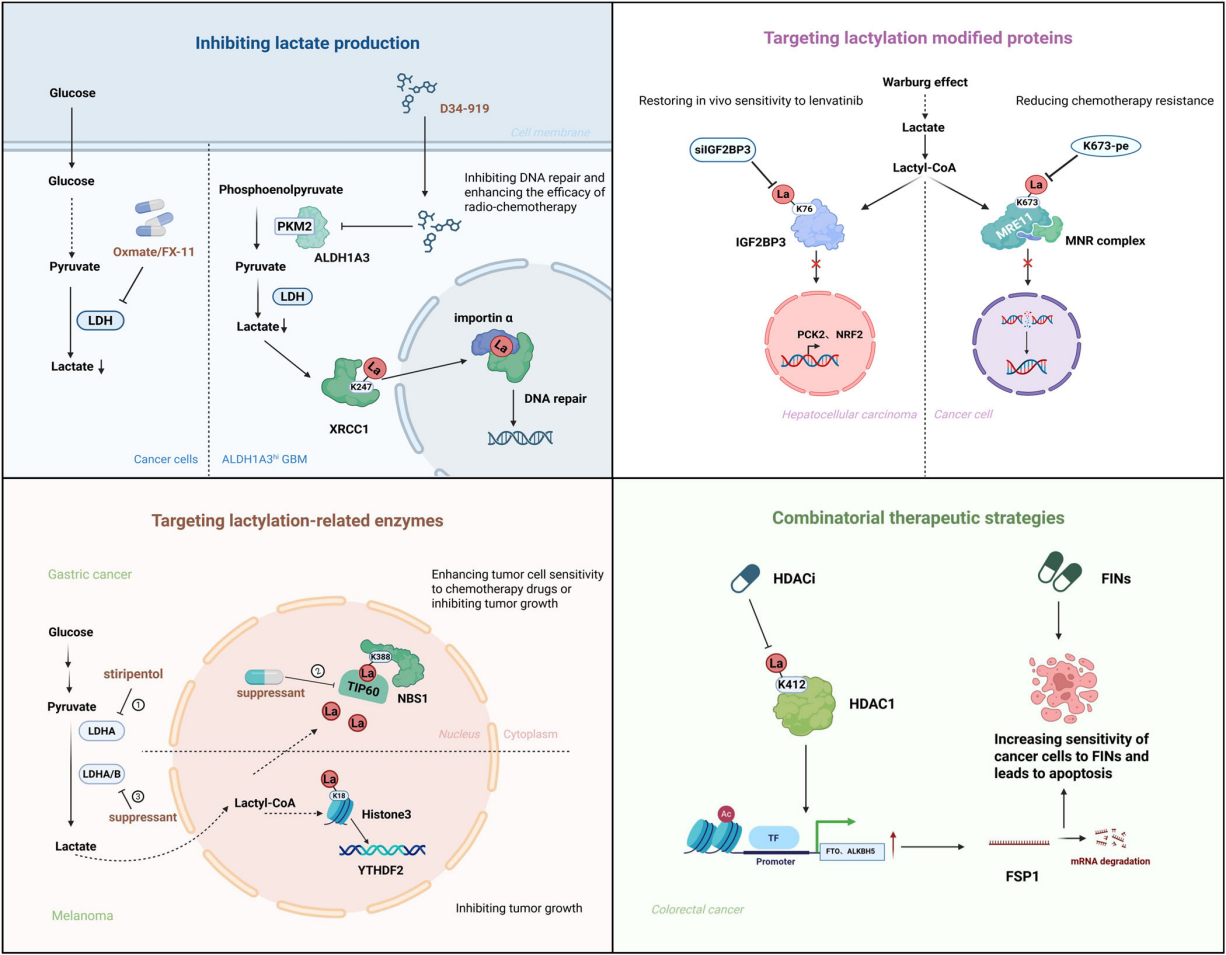
Potential therapeutic strategies targeting lactylation in cancer treatment. Targeting lactylation has emerged as a potential therapeutic strategy in cancer treatment. First, targeting lactylated proteins such as MRE11 and IGF2BP3 can enhance chemotherapy sensitivity. Second, inhibiting lactylation‐related enzymes, including lactyltransferases and lactate dehydrogenases, presents a viable approach for tumour suppression. For instance, inhibitors such as stiripentol can effectively reduce lactylation levels. Third, directly suppressing lactate production can lower lactylation levels, as demonstrated by lactate dehydrogenase (LDH) inhibitors such as Oxamate and FX‐11, or small molecules such as D34‐919, which disrupt the ALDH1A3‒PKM2 interaction to hinder tumour progression. Last, combinatorial strategies integrating lactylation‐targeted therapies with histone deacetylase (HDAC) inhibitors or metabolic modulators can further enhance therapeutic efficacy and overcome drug resistance. ALKBH5, AlkB homologue 5, RNA demethylase; ALDH1A3, aldehyde dehydrogenase 1 family member A3; FINs, ferroptosis inducers; FSP1, ferroptosis suppressor protein 1; FTO, fat mass and obesity‐associated gene; HDACi, histone deacetylase inhibitor; IGF2BP3, insulin‐like growth factor 2 mRNA‐binding protein 3; K673‐pe, K673‐peptide; La, lactylation; MNR complex, MRE11‒RAD50‒NBS1 complex; MRE11, meiotic recombination 11; NBS1, nijmegen breakage syndrome 1; NRF2, nuclear factor erythroid 2‐related factor 2; PCK2, phosphoenolpyruvate carboxykinase 2; PKM2, pyruvate kinase M2; siIGF2BP3, siRNA targeting IGF2BP3; TIP60, tat interacting protein 60 kDa; XRCC1, X‐ray repair cross complementing gene 1; YTHDF2, YTH N6‐methyladenosine RNA‐binding protein 2.

In addition, lactylation facilitates tumour growth and progression by modifying proteins such as eukaryotic translation elongation factor 1 alpha 2 (eEF1A2),^211^ restores homologous recombination repair pathways,^212^ and inhibits ferroptosis,^213^ thereby promoting chemoresistance, diminishing responsiveness to immune checkpoint inhibitors, enabling immune evasion, and ultimately correlating with unfavourable patient prognosis.^214^ Beyond these roles, lactylation has also been implicated in the regulation of oncogenic gene expression, such as minichromosome maintenance complex component 7 (MCM7),^215^ and the maintenance of cancer stemness through coordination with other PTMs.^216^ Given the extensive biological functions of lactylation, increasing attention has been directed towards its therapeutic potential in cancer, underscoring its emerging importance in tumour biology.^38^, ^217^, ^218^ Precisely because of its pivotal role in cancer progression and therapy resistance, a variety of strategies have been developed to target this modification. These approaches include inhibiting lactate production, directly targeting lactylated proteins, modulating lactylation‐regulating enzymes, and integrating lactylation‐targeted interventions with other therapeutic modalities (Figure 5).

One of the primary strategies focuses on inhibiting lactate production. LDH inhibitors, such as Oxamate and FX‐11, have demonstrated efficacy in reducing lactate production, and may therefore also mitigate lactylation modification.^219^, ^220^ In GBM with elevated ALDH1A3 expression, D34‐919 effectively disrupts the ALDH1A3‒PKM2 interaction, thus decreasing lactate accumulation and the lactylation of XRCC1 at K247, which in turn enhances the efficacy of radio‐chemotherapy.^154^ Another promising approach involves directly targeting lactylated proteins. For instance, lactylation of MRE11 at K673 promotes homologous recombination repair (HR), which contributes to chemotherapy resistance. Small molecule peptides specifically targeting MRE11 lactylation have shown potential in enhancing the efficacy of platinum‐based chemotherapies and poly ADP‐ribose polymerase (PARP) inhibitors.^212^ In HCC, excessive lactate production leads to the lactylation of insulin‐like growth factor 2 mRNA‐binding protein 3 (IGF2BP3), contributing to resistance to lenvatinib. Treatment with liposomal delivery of siRNA‐targeting IGF2BP3 or the glycolysis inhibitor 2‐DG restores in vivo sensitivity to lenvatinib.^221^ In addition, targeting enzymes involved in lactate production or lactylation modification offers a promising therapeutic approach. For instance, the lactylation of NBS1 at K388 has been found to be closely associated with poor prognosis and reduced chemotherapy sensitivity in various cancers.^80^ Studies suggest that inhibiting lactate dehydrogenase A (LDHA) activity or using stiripentol (a LDHA inhibitor used clinically for epilepsy treatment) can reduce lactate production, thereby lowering lactylation levels and inhibiting tumour progression. Furthermore, the research proposes that inhibiting the histone acetyltransferase TIP60 could reduce NBS1 lactylation, enhancing tumour cell sensitivity to chemotherapy drugs. In melanoma, co‐inhibition of LDHA and LDHB reduces lactate production, thereby decreasing H3K18 lactylation, suppressing YTHDF2 expression, and further inhibiting tumour growth.^222^ Finally, lactylation also interacts with other metabolic pathways to enable combinatorial therapeutic strategies. In colorectal cancer, histone deacetylase inhibitors (HDACi) enhance ferroptosis sensitivity by inhibiting lactylation modification of HDAC1, significantly improving the effectiveness of ferroptosis inducers (FINs) through targeting the HDAC1‐FTO/ALKBH5‐FSP1 axis.^223^

In conclusion, lactylation not only serves as a prognostic biomarker in cancer but also represents a potential therapeutic target. By specifically targeting lactylation‐modifying enzymes, lactylated proteins or combining metabolic and epigenetic interventions, new cancer treatment paradigms could be developed.

## 6 CONCLUSION

In conclusion, lactylation, a dynamic PTM, plays a critical role in coordinating metabolic states with cellular functions. Glycolytic enzymes, traditionally known for their metabolic functions, are now recognised for their involvement in mediating lactylation modification in cancer cells. As discussed in this review, due to their ability to generate lactate, glycolytic enzymes become key regulators of intracellular lactate levels.^31^, ^224^, ^225^ Given the heavy reliance of tumour cells on aerobic glycolysis, the modulation of lactylation by glycolytic enzymes is particularly prominent in cancer, thereby contributing to alterations in tumour biological behaviours.^139^, ^149^, ^156^, ^226^ Moreover, glycolytic enzymes not only influence lactylation processes but can themselves undergo lactylation, leading to modifications in their enzymatic activities and further impacting tumour progression.^20^, ^122^, ^123^ This intricate interplay between glycolysis and lactylation highlights the complexity of metabolic reprogramming in cancer and underscores lactylation as a crucial mediator linking metabolism to oncogenic signalling pathways.

Beyond cancer, lactylation is also implicated in various neurodegenerative and metabolic diseases, such as Alzheimer's disease, Parkinson's disease and Huntington's disease, indicating its broader role in pathological processes.^30^, ^227^, ^228^ In the TME, lactylation influences immune responses, inflammation and the interactions between tumour cells and immune cells. For instance, lactate accumulation promotes H3K18 lactylation, upregulating METTL3 in tumour‐infiltrating myeloid cells (TIMs), leading to immune suppression and poor prognosis in colorectal cancer patients.^229^ Lactylation also enhances the transcription of RUBCNL/Pacer, contributing to resistance to bevacizumab treatment.^230^ Additionally, lactylation at the K229 site of METTL16 has been identified in gastric cancer.^231^ These findings collectively position lactylation as an attractive therapeutic target for cancer intervention.

Glycolytic enzymes play a central role in the initiation and progression of tumours. Emerging evidence indicates that, beyond the aforementioned PTMs and enzymatic activation, genetic and epigenetic reprogramming of glycolytic enzyme–encoding genes represents a critical driving force underlying lactate accumulation, histone lactylation (Kla) and malignant transformation. Transcription factors such as hypoxia‐inducible factor‐1α (HIF‐1α) and MYC can bind to the promoters of GLUT1, HK2, LDHA and PKM2, thereby enhancing their transcriptional activity, accelerating glycolytic flux and promoting lactate production.^232^, ^233^ In PDAC, NUSAP1 forms a transcriptional complex with HIF‐1α and c‐Myc to upregulate LDHA expression, further facilitating lactate production and histone lactylation.^21^ Recent studies in breast cancer have identified two novel histone lactylation sites, H4K79la and H4K91la, which are enriched at the promoter regions of LDHA, PGK1 and HK1. These modifications reinforce glycolytic gene expression through a positive feedback mechanism.^234^ In CRC, METTL1‐mediated tRNA m^7^G modification enhances the translational efficiency of PKM, subsequently increasing H3K9la levels and facilitating immune evasion.^155^ Although current studies exploring the interconnection among three‐dimensional chromatin architecture, genomic alterations and lactylation remain limited, the existing evidence highlights the pivotal contribution of glycolytic gene regulation to the establishment of Kla, providing novel mechanistic insights into the metabolic–epigenetic crosstalk that governs tumour progression. However, despite growing research on lactylation, several challenges and limitations remain. First, the majority of lactylation sites have yet to be identified. Most studies to date rely on global detection methods such as mass spectrometry, and mechanistic investigations in vivo are still limited.^235^, ^236^ Second, although lactylation is generally reported to promote tumour progression, it may exert opposing effects depending on the context. As summarised in Table 1, the same glycolytic enzyme—for example, GLUT or aldolase—can exhibit distinct functions depending on the lactylation site and tumour type. Similarly, under different conditions, lactate in CD8⁺ T cells may suppress their activity, yet it can also enhance antitumour immunity by promoting effector functions in immune cells.^237^, ^238^ Accordingly, its effects must be interpreted with caution. Third, commonly used approaches to study lactylation, while valuable, have inherent limitations: they may alter intrinsic protein properties, induce non‐specific effects, or fail to accurately mimic physiological lactylation levels, making it difficult to establish causality.^58^, ^239^ Thus, the precise biological functions of lactylation remain to be fully elucidated. Moreover, research into how specific glycolytic enzymes, such as PGI, TPI and GAPDH, mediate lactylation in tumour cells remains limited. To date, there is still no conclusive evidence linking these enzymes directly to lactylation and cancer progression. In contrast, glycolytic enzymes such as HK and muscle‐type phosphofructokinase (PFKM) have been well characterised for their roles in lactylation‐driven tumour progression. Therefore, further investigation is warranted to elucidate whether PGI, TPI and GAPDH similarly contribute to the regulation of lactylation dynamics, either by modulating intracellular lactate levels or by serving as direct substrates for lactylation. Looking ahead, future research should focus on elucidating the genetic, structural and enzymatic determinants that govern the lactylation of glycolytic enzymes and their downstream effectors in tumour metabolism. The roles and mechanisms of gene modifications, expression regulation, three‐dimensional chromatin organisation, and genetic variations in glycolytic enzyme‐encoding genes during the process of lactylation remain to be fully elucidated. Integrating multi‐omics approaches, including genomics, proteomics and structural biology, may help uncover the molecular interplay between glycolytic metabolism and lactylation at single‐cell and spatial levels. At the same time, high‐resolution lactyl‐proteomic profiling with structural biology approaches, such as cryo‐electron microscopy and molecular dynamics simulations, may reveal the conformational features underlying specific enzyme–substrate interactions. The dynamic interplay between lactylation and other PTMs, including acetylation and phosphorylation, also warrants systematic investigation through multi‐omics and time‐resolved proteomic analyses to uncover potential crosstalk mechanisms that fine‐tune metabolic and signalling networks. In addition, applying single‐cell and spatial multi‐omics technologies to patient‐derived tumour specimens could help define lactylation‐dependent metabolic phenotypes associated with therapeutic resistance or immune modulation, thereby facilitating biomarker discovery. Ongoing efforts to develop selective chemical probes or small‐molecule inhibitors targeting lactylation‐related enzymes may be further leveraged in combination with other therapeutic strategies to enhance treatment precision, offering new opportunities for effective clinical intervention.^154^

Given the profound impact of lactylation on cancer biology, it presents an exciting opportunity for the development of novel cancer therapies that target metabolic reprogramming and immune modulation. As our understanding of lactylation's role in cancer continues to evolve, it may offer new therapeutic avenues for overcoming resistance to conventional treatments and improving patient outcomes.

## AUTHOR CONTRIBUTIONS

Lihua Wang conceived this study. Chenyuan Dai and Lihua Wang wrote the paper. All the authors read and approved the manuscript.

## CONFLICT OF INTEREST STATEMENT

The authors declare they have no conflicts of interest.

## ETHICS STATEMENT

Not applicable.

## Supporting information



Supporting Information

## Data Availability

Not applicable.
