# Supplementary material for: Glycolysis enzymes and cellular lactylation in tumour
Source: Clin Transl Med. 2026 Feb 18;16(2):e70549. doi: 10.1002/ctm2.70549 (PMC12914357; doi:10.1002/ctm2.70549)
Supplement: Supplementary file 1 — Supporting Information [file CTM2-16-e70549-s001.docx]

Supplementary Information from Section 3: Gene Knockout Studies in Lactylation Research

| **Target gene** | **Experimental System** | **Observed Effects** | **Reference** |
| --- | --- | --- | --- |
| BACH1 | Microglia and bach1 conditional knockout mice | Reduced microglia and astrocyte precursor populations | 107 |
| HK2 | Renal IRI mice | Abolished AST-120 protective effects | 108 |
| HK2 | Hepatic stellate cells and liver fibrosis mouse model | Reduced HSC activation and progression of liver fibrosis | 19 |
| NR4A3 | Mouse vascular calcification model | Reduced glycolysis, lactate production, and histone lactylation | 113 |
| ALDOA | Human liver cancer cell lines (HuH-7, SNU-449, MHCC-97L) and mouse model | Reduced proliferation, migration, invasion, and tumor growth | 121 |
| NEAT1 | Py8119 cell line, MCF7 and MMTV-PyVT mouse model | Reduced breast cancer growth, metastasis, and glycolytic flux | 146 |
| PKM2 | Endothelial cell-specific PKM2 knockout mice | Reduced osteogenesis | 159 |
| PKM2 | Cochlear organoids and pkm2 knockout mice | Impaired cochlear development | 160 |
| LDHA | MGC803 cell line | Reduced lactate production and NBS1 K388 lactylation | 80 |
